# Ultrasound-guided percutaneous microwave ablation assisted by three-dimensional visualization operative treatment planning system and percutaneous transhepatic cholangial drainage with intraductal chilled saline perfusion for larger hepatic hilum hepatocellular (D ≥ 3 cm): preliminary results

**DOI:** 10.18632/oncotarget.19275

**Published:** 2017-07-15

**Authors:** Xin Li, Jie Yu, Ping Liang, Xiaoling Yu, Zhigang Cheng, Zhiyu Han, Shaobo Duan, Jiasheng Zheng

**Affiliations:** ^1^ Department of Interventional Ultrasound, Chinese PLA General Hospital, Beijing, 100853, China; ^2^ Minimally Intervention Therapy Center of Liver Diseases and Oncology, Beijing You An Hospital, Capital Medical University, Beijing, 100069, China

**Keywords:** hepatic hilar hepatocellular carcinoma, ultrasound-guided percutaneous microwave ablation, three-dimensional visualization operative treatment planning system, percutaneous transhepatic cholangial drainage with intraductal chilled saline perfusion

## Abstract

To assess the clinical efficiency of ultrasound-guided percutaneous microwave ablation (US-PMWA) assisted by three-dimensional (3D) visualization operative treatment planning system and percutaneous transhepatic cholangial drainage with intraductal chilled saline perfusion (PTCD-ICSP) for larger hepatic hilum hepatocellular carcinoma (HH-HCC) (D ≥ 3 cm). The combination therapy was performed in 14 patients from Sep 2011 to May 2017. The major outcomes for assessment were biliary duct complications, local tumor recurrence, distant recurrence and overall survival rates. Median follow-up period was 26 months. The series of 3D visualization operative treatment planning, PTCD-ICSP and US-PMWA were successfully performed and complete ablation was achieved in all cases. The mean session for one tumor was 1.0 ± 0.4. The mean ablation time for per tumor was 1805 ± 567s. The saline volume used for the PTCD-ICSP was 250–450 ml per session. The 1-, 2-, and 3-year local tumor recurrence rates were 7.1%, 14.3%, and 35.7%, the 1-, 2-, and 3-year distant recurrence rates were 0%, 14.3%, and 28.6%, and 1-, 2-, and 3-year overall survival rates were 100%, 92.9%, and 71.4%, respectively. No severe complications related to ablation occurred.

**Conclusions:**

US-PMWA assisted by 3D visualization operative treatment planning system and PTCD-ICSP appears to be a safe, effective and innovative technique for management for larger HH-HCCs, which improved the prognosis.

## INTRODUCTION

Hepatocellular carcinoma (HCC) is a common malignancy with dismal prognosis, which listed the fifth most common cancer in men and seventh in women [1–3]. The morbidity and mortality rates is still increasing. Liver transplantation and surgical resection are the optimal treatments. However, the majority of HCC patients have a background of chronic liver diseases, especially cirrhosis. And a substantial proportion of patients are not eligible for candidate for lack of liver source and impaired liver function and/or multinodularity of tumor. In such patients, mini-invasive treatments have been applied widely in clinic, such as transcatheter arterial chemo-embolization (TACE), ethanol injection (EI), microwave ablation (MWA) and radiofrequency ablation (RFA) [4–5]. Among them, MWA is a widely accepted alternative to surgery in china because of its safety and high efficacy currently [6–7]. As for tumors located at hepatic hilum, treatment is quiet difficult for the close relationship with the surrounding structures such as biliary duct, portal vein, hepatic vein and artery, especially for diameter larger than 3 cm. Even surgical resection also faces difficulty because of the unique anatomy and high recurrence rate [8]. So image-guided percutanous ablation which has been increasingly accepted for its advantages of mini-invasion, favorable efficacy and good reproducibility would be a better choice [7, 9–10].

For larger HCC located at hepatic hilum (HH-HCC), the distance between the tumor and the surrounding structures such as biliary duct, portal vein, hepatic vein and artery is sometimes less than 5 mm. Blood vessels (portal vein, hepatic vein and artery) are thought to be relatively immune to the thermal injury because of the cooling effect of circulating fluid. But, the velocity of bile juice in the bile duct is much slower, and intrahepatic bile ducts near the tumor are prone to thermal injury during ablation. It was reported that bile duct dilatation affecting two or more subsegments should be regarded as a complication that may affect the prognosis and should be observed carefully [11]. How to protect biliary duct during ablation is crucially important. In 2007 and 2010, Ohnishi T and Ogawa T reported that intraductal chilled saline perfusion through a nasobiliary tube is a potential intervention method to prevent biliary injury in percutaneous RFA for HCC, respectively [12]. But the nasobiliary embeding might bring uncomfortable and inconvenience to patients during and after ablation. While percutaneous transhepatic cholangial drainage with intraductal chilled saline perfusion (PTCD-ICSP) could avoid the drawbacks and is performed easily.

Whether surgical resection or thermal ablation for larger HH-HCC, understanding the spatial anatomy relationship between the tumor and surrounding tissue is vital important, especially for image-guided percutanous ablation. The incidence of biliary stricture and bilomas is more inclined than smaller HH-HCCs which resulted from thermal damage to biliary duct. How to project the thermal plan precisely may reduce the biliary complication and achieve completely ablation.And percutanouse thermal ablation requires calculating thermal field distribution and determining how many probes to choose and how to implantation them, which is difficult to determine only depending on two-dimensional (2D) imaging, such as US, CT and MRI. Traditionally, the radiologist reconstruct a three-dimensional (3D) imaging in their own perception, but this is subjectively dependent on their spatial awareness and experience. The radiologist’s judgment is not consistent with the actual situation sometimes, and may lead to incomplete ablation or major complications. 3D visualization operation planning could display the location and spatial relationship of tumor with the surrounding structures, predict the time-temperature profile during ablation, improve the safety of ablation, reduce complications and promoter local tumor control [13–15]. In our preliminary study, 3D visualization operative treatment planning system was used in US-PMWA for liver cancer, which promoted precise therapy, decreased complications rate, ensured tumor-free safety margins and improved long-term survival outcomes for larger tumors [16–17]. So this novel technique may provide more information and valuable assistance for HH-HCC in US-PMWA.

Upon the above analysis, we performed this study to retrospectively analyzed the clinical efficiency of larger HH-HCC treated with US-PMWA assisted by 3D visualization operative treatment planning system and PTCD-ICSP.

## RESULTS

**Figure 1:**
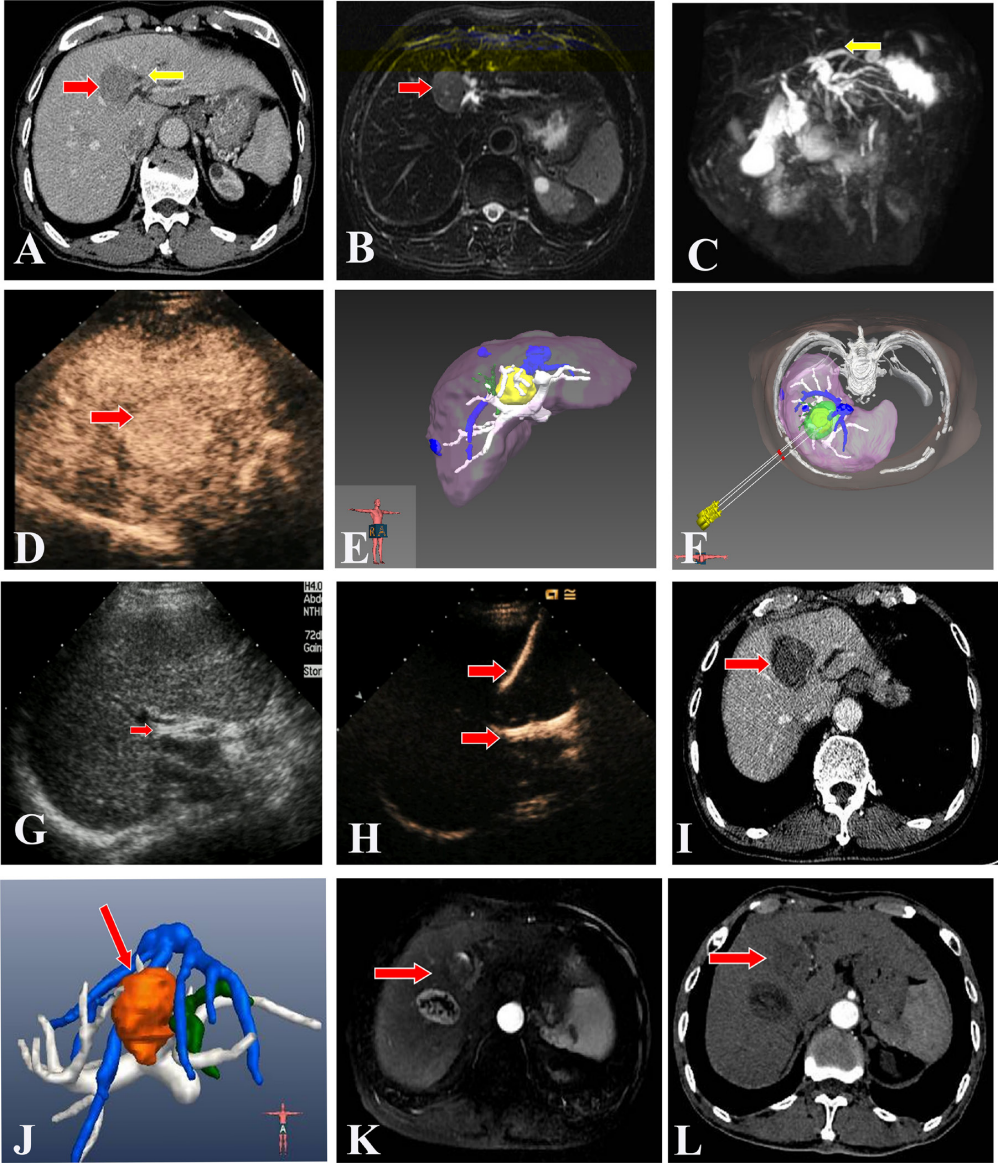
US-PMWA assisted by 3D visualization operation treatment planning and for a 74-year-old man with a larger HH-HCC (4.4 × 3.2 cm) **(A, B, C)** Pre-operation CT imaging showed a larger HH-HCC (red arrow). The left hepatic lobe intrahepatic biliary duct expansion (yellow arrow). Pre-operation magnetic resonance cholangiopancreatography (MRCP) imaging showed a larger HH-HCC (red arrow) and intrahepatic biliary duct expansion in the left hepatic lobe (yellow arrow). **(D, E, F)** Pre-operation CEUS imaging showed a larger HH-HCC with hyper-enhancement in artery phase (red arrow). Pre-operation 3D visualization treatment planning system showed the location of the tumor and the close relationship with the surrounding structures, and three antennas were need to ablate the tumor completely and safely. (Yellow: tumor; White: hepatic portal vein; Green: biliary duct; Blue: hepatic vein) **(G, H, I)** A PTCD tube was inserted percutanously into the left hepatic biliary duct before PMWA to prevent thermal injury of the duct, which was verified by CEUS examination. A significant ablation effect on the tumor was obtained by a series of PMWA, as shown on a contrast-enhanced CT image. (red arrow). **(J, K, L)** Post-operation 3D visualization treatment planning system evaluated the ablation effect with completely ablation without biliary duct injury. The contrast-enhanced MRI images 1 year after ablation showed complete tumor necrosis and the ablation zone shrunk gradually (red arrow). While there was a intrahepatic distant recurrence beside the primary tumor at 1 year after ablation and recieved another procedure. In Figure L, there was no local progress in the two ablation zones on contrast-enhanced CT images at 2 years after first ablation. (Brown: ablation zone; White: hepatic portal vein; Green: biliary duct; Blue: hepatic vein).

All patients and tumors characters, ablation data was listed in Table 1. All HH-HCC were successfully performed contrast-enhanced CT/MRI scanning with slice thickness and 3D visualization operation treatment planning before ablation (Figure 1). PTCD-ICSP and real-time thermal monitoring system were also successfully implemented during ablation with one time. All patients showed good tolerance. There were no complications during any of the US-PMWA sessions. All HH-HCC were completely ablated according to the 3D visualization operation treatment planning (Figure 1). The mean insertion number was 5.2 ± 1.0 (ranged, 2–8). Six (42.9%) patients underwent TACE 1 month before ablation. Among the 14 tumors, complete ablation were achieved in 12 (85.7%) patients by one session and 2 (14.3 %) patients by two, who had residual unablated tumor at treated site adjacent to portal vein and received another ablation. The mean session for one tumor was 1.0 ± 0.4 sessions. The mean time of ablation for per tumor was 1805 ± 567s (ranged 960–2880s). The saline volume used for the PTCD-ICSP was 250–450 ml (median 340 ml) per session.

**Table 1:** The characters and parameters of patients, tumors and ablations

No	Age/sex	Size (cm)	Location	TA	DD	Child-P	IN	AT(s)	SV(ml)	session	Follow-up (m)	LR	DR	Died	PT
1	77/F	3.1	S5	B	M	A	4	1440	300	1	27	No	No	No	No
2	72/M	3.2	S8	B	M	A	2	1260	280	1	52	No	Yes	Yes	No
3	68/F	3	S8	C	M	A	2	960	250	1	49	No	No	No	No
4	75/M	4.4	S8	No	M	B	4	1800	330	1	25	No	No	No	TACE
5	72/M	6.8	S5/8	B	H	B	8	2880	450	2	19	Yes	Yes	Yes	TACE
6	58/M	4.6	S5	B	M	A	4	2000	350	1	26	No	No	No	No
7	61/M	5.4	S5	B	M	B	4	2680	430	2	27	Yes	No	No	TACE
8	75/F	3.6	S5	C	M	A	4	1360	300	1	26	No	No	No	No
9	67/M	3.4	S4	B	M	B	4	1260	290	1	25	No	No	No	No
10	57/M	4.1	S8	B	M	B	4	1920	350	1	34	Yes	Yes	Yes	No
11	63/F	3.6.	S4	B	H	B	4	1880	350	1	23	No	No	No	TACE
12	74/F	3.2	S4/5	C	H	A	2	1480	310	1	25	No	No	No	No
13	70/M	6.3	S5/8	B	H	A	6	2460	410	2	24	Yes	Yes	Yes	TACE
14	61/F	3.7	S5	No	M	B	4	1890	360	1	44	Yes	No	No	TACE

*S: Section, TA: Type of hapatitis; DD: differentiated degree; M: Middle; H:High; IN: Insertions number; AT: Ablation time; SV: saline volume; LR: Local recurrence; DR: Distant recurrence; PT: Previous therapy; TACE: Transcatheter arterial chemo-embolization.

Median follow-up period was 26 months (ranged 19–52 months). Five (35.7%) patients had progression at treated site adjacent to portal vein at 3, 14, 26, 32 and 28 and months after ablation. One (7.1%) patients had progression to tumor thrombus in right portal vein at 5 months after ablation. Four (28.6%) patients with distant metastasis to brain, lung and bone at 13, 18, 26 and 43 months after ablation. Four (28.6%) patients died from brain, lung, bone metastases and cardiac failure at 19, 24, 34 and 52 and months after ablation, respectively. The 1-, 2-, and 3-year local tumor recurrence rates were 7.1%, 14.3%, and 35.7%, the 1-, 2-, and 3-year distant recurrence rates were 0%, 14.3%, and 28.6%, and 1-, 2-, and 3-year overall survival rates were 100%, 92.9%, and 71.4%, respectively. No severe complications (biliary fistula, biliary stenosis, obstructive jaundice and abscess) related to ablation occurred. The common side effects such as transaminase and bilirubin elevation (14 patients, 100%), fever (6 patients, 42.8%), abdominal pain (7 patients, 50%) and hemoglobinuria (5 patients, 41.7%) ocurred. All of the side effects were disappear with conservative treatment in 24–72 hours.

## DISCUSSION

Percutaneous image-guided thermal ablation has been reported to be an effective technique and yield promising prognosis in HCC and widely applied in clinic for minimal invasive, well therapeutic effect and no radiation and chemotherapy side effects. MWA and RFA methods are the two main choices. Although RFA is a safe procedure with low morbidity [18–19], there have been several reports of biliary injury after this treatment for HH-HCC. Biliary stricture, bilomas and hemobilia are the main complications which may result to liver abscess, even death. The rate of biliary injury after thermal ablation for HH-HCC varies differently (0.3–17%) [12, 20–21]. For larger tumors, MWA uses electromagnetic energy to rapidly rotate adjacent polar water molecules, which shows several theoretical advantages over RFA in consistently. Higher intratumoral temperatures, larger ablation volumes, faster ablation times, less dependency on the electrical conductivities of tissue and energy delivery which promote the thermal efficiency and coverage thermal field [22–23]. So in this study, MWA was used to treat larger HH-HCC may show better tumor local control and long term prognosis. But the biliary injury maybe more inclined to occurred in MWA treatment for HH-HCC [24]. Therefore, tumors located near the hepatic hilum is generally considered to be a contraindication for thermal ablation.

To overcome these limitations, biliary stent implantation and intraductal chilled saline perfusion during ablation were the main protective measures. While intraductal chilled saline perfusion through a nasobiliary tube was widely used in clinic and achieved good clinical efficiency [12, 25]. For PTCD was an simple and easy to operate in clinic, we choose PTCD-ICSP as substitution for ICSP through a nasobiliary tube in this study. In these case, all patients showed good tolerance. After PTCD-ICSP was embedded, chilled saline was infused constantly through the tube during ablation and till 2 hours after ablation which further to cool the residual heat. Follow-up imaging during the subsequent follow-up period showed no evidence of injury to the biliary ducts. The theory speculation was that the infusion of chilled saline might create a pseudo “heat sink” effect resulting in protection of the wall of the bile duct by causing heat loss at the margin of the ablation zone to the liquid “flowing” at a lower temperature in the adjacent biliary ducts [26].

Preoperative treatment planning as the first step in the thermal ablation process acts to lower the rate of complications, ensure tumor-free safety margins after ablation, and improve long-term survival [16]. As the proverb goes “A good beginning is half done”. Good preoperation treatment planning is crucial for successfully ablation. Treatment for HH-HCC is challenging because of their location and complex anatomy, especially for larger ones. Sometimes the close relationship between tumor with one and/or two even more important structures (bile duct, portal vein, hepatic vein and artery). It is difficult to display on 2D imaging and reconstruct in the operators’ perception subjectively. 3D visualization operative treatment planning system has been used in surgery in recent years, but seldom in image-guided percutaneous thermal ablation [15]. In 2012, a original 3D-visualization operative treatment planning system used for MWA guided by ultrasound was applied in HCC ablation, which promoted the rate of complete ablation by one session and long term efficacy significantly [16]. In this study, 3D visualization operative planning not only precisely display the location and the relationship with the surrounding structures of the tumor, and calculate the thermal field of multi-antenna and multi-point ablation. Meanwhile, the precisely demonstration the expansion of biliary ducts could be beneficial to the performance of PTCD-ICSP. So in the study, the operation of PTCD-ICSP and thermal monitoring needle were carried out with one time, which shorted the operation time and reduced the complications.

With the assistance of 3D visualization operation treatment planning system and PTCD-ICSP for larger HH-HCC, complete ablation were achieved in 12 (85.7%) patients by one session and 2 (14.3%) patients by two, who had residual tumor at treated site adjacent to portal vein and received another ablation. Maybe the reason might be worrying to damage portal vein and not achieved completely ablation. And during the follow-up period, the tumor progression were also adjacent to portal vein. The two phenomena demonstrated that if effective protective measures were carried out for portal vein, there were no residual tumor and progression. One patients had progression to tumor thrombus in right portal vein at 5 months after ablation, which may related with tumor progression. The 1-, 2-, and 3-year local tumor recurrence rates were 7.1%, 14.3%, and 35.7% and the 1-, 2-, and 3-year distant recurrence rates were 0%, 14.3%, and 28.6%, respectively. which reduced obviously. And , the 1-, 2-, and 3-year overall survival rates were 100%, 92.9%, and 71.4%, respectively. In the short-time and long-time follow-up period, there was no severe complications such as biliary fistula, biliary stenosis, obstructive jaundice and abscess occurred. All of the results showed that the assistance of 3D visualization operation treatment planning and PTCD-ICSP overcame the treatment limitations of US-PMWA for larger HH-HCC and improved the clinical efficiency.

While some limitations of the study were listed as follows. Firstly, this study was a preliminary study and the sample is smaller; a further study with larger sample, randomized controlled study and longer follow-up period may be mandatory. Secondly, the residual tumor and progression both adjacent to portal vein, a effective method should think out to solve the problem. Thirdly, the operators were with more than 5 years ablation experience, and more intelligent 3D visualization operation planning platform were needed to further exploit for younger operators. And a multi-centre study would be more convincing.

## MATERIALS AND METHODS

### Patients and tumors

This retrospective study was approved by our Institutional Ethics Committee, and written informed consent for the procedure was obtained from each enrolled patient in the study. From Sep 2011 to Mar 2017, 14 patients with 14 larger HH-HCCs were enrolled and underwent US-PMWA assisted by 3D visualization operation treatment planning and PTCD-ICSP at our department. There were 6 female and 8 male patients, with average age 68 + 7 years (ranged 57–75 years). Average HH-HCC maximum diameter was 4.2 ± 1.3 cm (ranged, 3.0–6.8 cm). The maximum distance between the tumor with secondary bile duct was less than 5mm. The diagnosis of HCC was based on histological evidence from needle biopsy specimens, while in the absence of histological results, two contrast-enhanced imaging showing typical features and medical history of HCC [2, 7]. All patients were closely followed up until Mar 2017. All medical and imaging records were reviewed and analyzed.

### Pre-ablation examination

The inclusion criteria for this study were as followed: 1) non-resectable tumors or patient refusal to undergo surgery; 2) single HCC lesion ≤ 8 cm; 3) absence of portal vein and/or biliary duct thrombosis or extrahepatic metastasis; 4) prothrombin time < 25 s; 5) prothrombin activity > 40%; 6) platelet count > 60 cells × 10^9^/L; 7) serum total bilirubin concentration < 40 umol/L. The exclusion criteria were listed as followed: 1) severe cardiopulmonary disease; 2) serious renal function failure; 3) severe liver function failure, such as uncontrollable ascites, hepatic encephalopathy, serious esophageal gastric varices; 4) active severe infection; 5) with a prior history of bilioenteric anastomosis were excluded for this condition was a risk factor for hepatic abscess formation after PMWA.

### 3D visualization operation treatment planning

All patients had undergone conventional ultrasound, CEUS, CT/MRI to delineate the target tumor within 7 days before ablation. Our group original developed 3D visualization operation planning software that includes tumor segmentation, access-path planning, simulation of ablation volume, and visualization of both the patient anatomy and the planned strategy [16–17]. Contrast-enhanced CT/MRI imaging was import into the EFILM software, and the data was analyzed by 3D visualization platform. Tumor volume calculation, stereo display of the relationship between tumor and surrounding structures, planning the number, embedding path and angle of antennas, simulation the thermal field distribution and pre-assessment ablation effect were demonstrate precisely by 3D visualization operation treatment planning system.

The operation planning of US-PMWA should abide by the following principles: (1) for the distance between the tumor boundary and bile duct was equal to or larger than 5 mm, expanded ablation was applied; or else conformal ablation was applied. Expanded ablation is covering the entire tumor plus a predefined safety margin (3–5 mm) as much as possible. Conformal ablation is covering the entire tumor only; (2) avoiding ablation of any vital structures, especially secondary biliary duct in this study; (3) minimizing the number of antenna insertions and ablation points; (4) avoiding puncture of critical structures along the path of insertion.

### PTCD-ICSP and US-guided PMWA

The treatments were performed on an in-patient basis. The PTCD was performed according to the standard method in the enrolled patients before ablation [27]. For the biliary tube drainage, we used a 6 or 8.5F drainage catheter (COOK, Winston-Salem, NC, USA) with more side-holes. The tip of the tube was positioned either in the subsegmental branch or the hepatic duct close to the lesion. we confirmed the tube was in the proper position by injecting an ultrasound contrast agent (Sonovue BR1; Bracco SpA; Milan, Italy) into the tube before ablation, and by observing the flow in the biliary duct surrounding the tumor. Frozen saline was thawed just before ablation, and was perfused during the whole ablation procedure through the tube by drip infusion with the speed of 0.2 ml/s, then the speed was changed to 0.05 ml/s until 2 hours after ablation.

A microwave system (KY-2000, Kangyou Medical, Nanjing, China) comprising a MW generator, flexible coaxialcable and cooled-shaft antenna. The generator can drive two antennas simultaneously with the capability of producing 1–100 W of power at 2450 MHz. The 15 G cooled-shaft antenna is coated with Teflon to prevent adhesion with dual channels inside the antenna shaft, through which distilled water circulated continuously by a peristaltic pump could cool the shaft to prevent overheating. There were two types of cooled-shaft antenna with 1.1 cm and 0.5 cm active tip. The 21G thermocouple needles were equipped on the MWA system which are easily seen by US. The tip of thermosensor was made of iron constant which is both accurate and sensitive to temperature, while not influenced by the electromagnetic field of MW.

US-guided biopsy was performed firstly using an automatic biopsy gun with an 18 G cutting needle under local anesthesia with 1% lidocaine before ablation; two or three punctures were performed. Subsequently, the antennas were percutaneously inserted into the tumor and placed in the desired location by US guidance. We used two antennas at one insertion and repeated according the diameter and the location of tumor. General anesthesia (propofol, 6–12 mg/kg per hour; ketamine, 1–2 mg/kg) was employed after placement the antennas properly, and ablation was implemented. A 21G thermocouple was placed percutaneously at a designated location (the distance between the tumor with secondary bile duct was less than 5 mm) to monitor temperature in real time [28]. The temperature was kept at 50°C–54°C for no longer than 3 minutes, with intermittent emission of microwaves [29]. When the hyperecho overlapped the whole lesion, the antennas were withdrawn. Then CEUS was performed 1 h after ablation to evaluate ablation effect. If there were no residual unablated tumor, the tube was removed 2 h hours after ablation. Otherwise, additional ablation was performed immediately until there was no enhancement in the whole tumor. At the meantime, CEUS via the PTCD tube before the removal of the tube did not show biliary injury in any of the patients. During the process, the needle tracks were routinely cauterized to avoid bleeding and tumor seeding. The insertion number, treatment time and saline volume were recorded. Insertion number was defined as the total number of antenna placements for each tumor until the tumor was completely ablated. Treatment time was the sum of ablation time of each antenna. The saline volume used for the PTCD-ICSP was the sum of volume used during the ablation.

### Post-ablation imaging follow-up

After ablation, patients were closely monitored for possible complications such as biliary fistula, biliary stenosis, obstructive jaundice and abscess. Technique effectiveness and local tumor recurrence were evaluated. Contrast-enhanced CT/MRI and CEUS was performed 3 days after the ablation to evaluate technique effect. Complete ablation was defined as a lack of enhancement of the entire tumor on images. If residual tumor was found on imaging, a further ablation session under CEUS guidance was performed in 7 days, or else patients entered the follow-up period, which consisted of contrast-enhanced CT/MRI and CEUS at 1, 3 and every 3 months intervals thereafter after ablation until Mar 2017. Local tumor recurrence was defined as the reappearance of tumor enhancement within or adjacent to the ablation zone.

### Statistical analysis

SPSS 17.0 for windows (SPSS Inc, Chicago, IL, USA) was used for data analysis. Continuous data were presented as means ± standard deviations (SD). *P* < 0.05 was considered statistically significant.

## CONCLUSIONS

US-PMWA assisted by 3D visualization operation treatment planning system and PTCD-ICSP appears to be a safe and effective technique for the management for larger HH-HCC (D ≥ 3 cm). 3D visualization operation treatment planning system provides spatial structure of the tumor and the surrounding structures, and PTCD-ICSP is a effective method to protect bile duct for US-PMWA in the patients with HH-HCC. The combination treatment enlarged indications of US-PMWA and improve the clinical efficiency, therefore, a larger number of patients will be able to benefit from this therapy.

## Abbreviations

(US-PMWA): US-guided percutaneous microwave ablation
(PTCD-ICSP): percutaneous transhepatic cholangial drainage with intraductal chilled saline perfusion
(HH-HCC): hepatic hilum hepatocellular carcinoma
(TACE): transcatheter arterial chemo-embolization
(EI): ethanol injection
(MWA): microwave ablation
(RFA): radiofrequency ablation
(CT): computed tomography
(MRI): magnetic resonance imaging
(PET): positron emission tomography
(2D): two-dimensional
